# Comparative Analysis of the Volatile Components of *Agrimonia eupatoria* from Leaves and Roots by Gas Chromatography-Mass Spectrometry and Multivariate Curve Resolution

**DOI:** 10.1155/2013/246986

**Published:** 2013-10-27

**Authors:** Xiao-Liang Feng, Yun-biao He, Yi-Zeng Liang, Yu-Lin Wang, Lan-Fang Huang, Jian-Wei Xie

**Affiliations:** ^1^School of Chemical and Material Engineering, Quzhou College, Quzhou 324000, China; ^2^Changde Institute for Food and Durg Control, Changde 415000, China; ^3^College of Chemistry and Chemical Engineering, Central South University, Changsha 410083, China

## Abstract

Gas chromatography-mass spectrometry and multivariate curve resolution were applied to the differential analysis of the volatile components in *Agrimonia eupatoria* specimens from different plant parts. After extracted with water distillation method, the volatile components in *Agrimonia eupatoria* from leaves and roots were detected by GC-MS. Then the qualitative and quantitative analysis of the volatile components in the main root of *Agrimonia eupatoria* was completed with the help of subwindow factor analysis resolving two-dimensional original data into mass spectra and chromatograms. 68 of 87 separated constituents in the total ion chromatogram of the volatile components were identified and quantified, accounting for about 87.03% of the total content. Then, the common peaks in leaf were extracted with orthogonal projection resolution method. Among the components determined, there were 52 components coexisting in the studied samples although the relative content of each component showed difference to some extent. The results showed a fair consistency in their GC-MS fingerprint. It was the first time to apply orthogonal projection method to compare different plant parts of *Agrimonia eupatoria*, and it reduced the burden of qualitative analysis as well as the subjectivity. The obtained results proved the combined approach powerful for the analysis of complex *Agrimonia eupatoria* samples. The developed method can be used to further study and quality control of *Agrimonia eupatoria*.

## 1. Introduction

*Agrimonia eupatoria*, one of Rosaceae plant family, mainly locates in Zhejiang, Yunnan, Guangdong, Guangxi, and other places of China [1]. As a traditional Chinese medicine (TCM) listed in the Chinese Pharmacopoeia, *Agrimonia eupatoria* has long been used to cure many diseases, such as tumors, Meniere's syndrome, and trichomonas vaginitis [2, 3]. It also has the function of antitumor antidiabetic, hemostatic, and antibacterial [4–6]. Although *Agrimonia eupatoria* contains up to tens or even hundreds of compounds, only a limited number of compounds, such as agrimony, agrimony lactone, tannin, flavonoids, glycosides, and the volatile components, might be the main active components, which are responsible for pharmaceutical or toxic effects [7, 8]. To ensure the reliability and repeatability of pharmacological and clinical research and understand their bioactivities and possible side effects of active compounds, it is necessary to study all of the phytochemical constituents of botanical extracts and develop a method for quality control of *Agrimonia eupatoria*. For example, the volatile constituents are known to exhibit pharmacological and biological activity, and it is used for sterilization and antibacterial active role [8]. Thus the analysis of the volatile ingredients in *Agrimonia eupatoria* is very important.

As for the analysis of the volatile compounds in *Agrimonia eupatoria*, only a few reports have been seen in the literature [9, 10]. They are usually performed with gas chromatography (GC) and gas chromatography-mass spectrometry (GC-MS), which are based on gas chromatographic retention indices or MS for qualitative and quantitative analysis. However, because the composition of *Agrimonia eupatoria* is very complicated and the contents of many important volatile components in *Agrimonia eupatoria* are very low, suitable sample-preparing methods are necessary before detection by GC-MS, such as steam distillation [9]. Although preparing methods are used for the analysis process of the complicated *Agrimonia eupatoria* samples, it is still impossible to obtain complete separation of all the volatile chemical constituents of *Agrimonia eupatoria.* In these general GC or GC-MS reports, it is difficult to assess the purity of chromatographic peaks and the peak inspected as one component may be a mixture of several components. The results obtained by these methods which have been mentioned above would be questionable. Fortunately, with the development of hyphenated instruments, multidimensional data revealing the compositions of samples can be obtained from GC-MS, HPLC-DAD, and so on. Then, many associated chemometric methods [11–18], which can be used to resolve multidimensional data, have been developed. Thus, more information for chemical analysis both in chromatographic separation and in spectral identification can be obtained, which makes it possible to interpret these complex systems.

On the other hand, there may be some sameness and differences to exist in *Agrimonia eupatoria* from different plant parts. To find the pharmacological active components that exist in essential oils exactly, it is important that the method for the detailed study of the components in *Agrimonia eupatoria* from different plant parts, such as the root and leaf, was established. 

In this paper, two chemometrics methods, subwindow factor analysis [13] and orthogonal projection resolution (OPR) [14], were used to analyze the volatile constituents of *Agrimonia eupatoria* from different plant parts for the first time. The volatile components of* Agrimonia eupatoria* from two different plant parts were extracted with water distillation and subjected to GC-MS analysis. Firstly, the qualitative and quantitative analysis of volatile components in the main root of *Agrimonia eupatoria* was completed with the help of subwindow factor analysis. Secondly, the common peaks in leaf of *Agrimonia eupatoria* were extracted with orthogonal projection method. At last, to those constituents in leaf, which were not identified with OPR method, the qualitative analysis was also performed with subwindow factor analysis. Then, a simple and reliable combined approach for the systematic study of the volatile constituents in the main root and leaf *Agrimonia eupatoria* was developed. Not only more information was obtained, but also the reliability of components was improved. The obtained results can provide foundation for further development of fingerprint and quality control of *Agrimonia eupatoria*. 

## 2. Theory and Method

### 2.1. Subwindow Factor Analysis (SFA)

The detailed process of SFA has been described in the literature [13]; here only a brief depiction of the method is given.

According to the Lambert-Beer Law, a two-dimensional data **X**_*m*×*n*_ produced by hyphenated instruments can be expressed as the product of two matrices as follows:

(1) $$\begin{array}{c} X_{m\times n}=C_{m\times p\;\;}S_{n\times p}^{T}+E, \end{array}$$

where **X**_*m*×*n*_ denotes response matrix representing *p* components of *m* spectra measured at regular time intervals and at *n* different wavelengths or mass-to-charge ratios. Matrix **C** is the pure composed of *p* columns, each one describing the chromatographic concentration profile of a pure chemical species. Similarly, the matrix **S** ^*T*^ consists of *p* rows corresponding to pure spectra of the chemical species. Matrix **E** denotes measurement noise. The superscript *T* represents the transpose of matrix.

It is crucial to identify left and right subwindows of SFA. In the former an interfering compound starts to elute before the analyte appears in a chromatogram to the left of the analyte, and in the latter another interference continues to elute after the analyte has stopped eluting. The rank analysis can provide the number of chemical components of the left and right ones, say *m*_1_ and *m*_2_, respectively. And the number of components in the combination of left and right ones is *m*_1_ + *m*_2_ − 1, since the analyte is common to both. One may then find an orthogonal basis {**g**_1_, **g**_2_,…**g**_*m*1_} spanning the spectral subspace of the left subwindow and a similar basis {**f**_1_, **f**_2_,…**f**_*m*2_} spanning that of the right subwindow, corresponding to matrices **G** and **F**, by means of singular-value decomposition. The common spectral vector **v** to both subspaces can be written as linear combinations of both sets of basis for an ideal case:

(2) $$\begin{array}{c} v=Ga,\\ v=Fb. \end{array}$$

Under the conditions, **a** ^*T*^ **a** = **b** ^*T*^ **b** = 1. In reality, **G** **a** and **F** **b** are not identical on account of interference from noise and background and so forth. And we search for vectors **a** and **b** which minimize the squared norm:

(3) $$\begin{array}{c} N={||Ga-\;Fb||}^{2}=a^{T}G^{T}Ga+b^{T}F^{T}Fb-{2a}^{T}G^{T}Fb. \end{array}$$

Since **G** ^*T*^ **G** = **I** *m*_1_ and **F** ^*T*^ **F** = **I** *m*_2_ (unit matrices of dimension *m*_1_ × *m*_1_, and *m*_2_ × *m*_2_, resp.), we obtain

(4) $$\begin{array}{c} N=2-2a^{T}G^{T}Fb. \end{array}$$

Here, if **a** and **b** are the left and right singular vectors, respectively, associated with the first largest singular value *d*_1_ of the matrix **G** ^*T*^ **F** inserting this result in (3), we again obtain

(5) $$\begin{array}{c} N=2\left(1-d_{1}\right). \end{array}$$

The singular values *d*_*i*_ of the matrix **G** ^*T*^ **F** are in the range 0 ≤ *d*_*i*_ ≤ 1, and the larger the value of *d*_1_ is, the closer the agreement between **G** **a** and **F** **b** is. Thus, it makes a spectrum control possible that only vector **v** is common for the left and right two windows. Therefore, one can directly obtain component spectra. If all the pure spectra are available, the concentration profiles could be achieved by using prior information of spectra and linear regression:

(6) $$\begin{array}{c} C=XS{\left(S^{T}S\right)}^{-1}\;. \end{array}$$

It is worth noting that if there is no common vector the largest singular value *d*_1_ will be significantly less than 1. On the other hand, if there are two or more common vectors, the second singular value *d*_2_, even the third one, or more will also be close to 1. In both cases, one lacks information for the unique identification of the spectral vector **v**. 

### 2.2. Orthogonal Projection Resolution (OPR)

Because it has been described in detail in the literature [14], a brief depiction of orthogonal projection resolution was given as follows.

The orthogonal projection matrix **P**_*i*_ on to the complementary subspace *X*_*i*_^*T*^  is defined as:

(7) $$\begin{array}{c} P_{i}=I-X_{i}^{T}{\left({\text{X}}_{i}^{T}\right)}^{+}, \end{array}$$

where the superscript + denotes the Moore-Penrose pseudoinverse and **I** designates the identity matrix. *X*_*i*_^*T*^ represents different submatrices, which are a series of fixed size window matrices moving along the chromatographic direction.

Assume that the subspace spanned by the mixture spectra in *X*_*i*_^*T*^ is **M**. The residue vector **r**_*i*_ is given by

(8) $$\begin{array}{c} r_{i}=P_{i}v_{a}, \end{array}$$

where **v**_*a*_ denotes the spectrum of certain component that is resolved by the SFA and **r**_*i*_ is the projection of **v**_*a*_ on the orthogonal complementary subspace of **M**. Therefore, one has the length of the residue vector:

(9) $$\begin{array}{c} {\text{re}}_{i}={||r_{i}||}^{2}\;\left(i=1,2,\ldots ,m-w+1\right), \end{array}$$

where ||**r**_*i*_|| designates the Euclidean norm of the vector, *m* is the number of measured chromatographic points, and *w* is the size of window.

Plotting the valve of re_*i*_ versus the index *i*, one can obtain a graph. Here we call it spectrum projection graph, which can tell us whether the component is present or absent and where the component elutes. Suppose that the submatrix **X**_*i*_ contains component *a*. Then the spectrum of component *a* is in the subspace **M** spanned by the mixture spectra in *X*_*i*_^*T*^; hence the length of the residue vector will be close to zero. Otherwise, if the component a is not in the submatrix *X*_*i*_^*T*^, then re_*i*_ will have a relatively large valve.

## 3. Experimental

### 3.1. Instruments

GC-MS was performed with Shimadzu GCMS-QP2010 instrument. The volatile constituents in both the main root and leaf of *Agrimonia eupatoria* were separated on a 30 m × 0.25 mm I.D. fused silica capillary column coated with 0.25 *µ*m film OV-1.

### 3.2. Materials and Regents

The main root and leaf of *Agrimonia eupatoria* were obtained from a Zhejiang herbs nursery and were identified by a researcher from Institute of Materia Medica, Hunan Academy of Traditional Chinese Medicine and Materia Medica. Ether and anhydrous sodium sulfate were of analytical grade.

### 3.3. Extraction of the Volatile Components

The main root and leaf of *Agrimonia eupatoria* were dried at 40°C for about 40 min. Some 400 g dried *Agrimonia eupatoria* and 1200 mL distilled water were premixed, then placing them into a standard extractor. The mixture was allowed to stand for 30 min at room temperature before extracting the essential oil. Essential oil was obtained by the standard extracting method for essential oil in TCMs according to the Chinese Pharmacopoeia [19]. Effluent was extracted with ether, and the ether was removed by blowing with nitrogen under low temperature. The obtained essential oils were dried with anhydrous sodium sulfate and stored in the refrigerator at 4°C prior to analysis. 

### 3.4. Detection of Essential Oil

GC-MS was used to obtain chromatograms of essential oils. The oven was held at 70°C for 1 min during injection, then temperature programmed at 3°C min^−1^ to a final temperature of 210°C, and held for 5 min. Inlet temperature was kept at 270°C all the time. 1.0 *µ*L volume of essential oil was injected into the GC. Helium carrier gas at a constant flow-rate of 1.0 mL·min^−1^ and a 5 : 1 split ratio were used simultaneously. Mass spectrometer was operated in full scan and electron impact (EI+) modes with an electron energy of 70 eV; interface temperature: 270°C; MS source temperature: 230°C; MS quadrupole temperature: 160°C. In the range of *m*/*z* 30 to 500, mass spectra were recorded with 3.12 s·scan^−1^ velocity. 

### 3.5. Data Analysis

Data analysis was performed on a Pentium based IBM compatible personal computer. All programs of the chemometrical resolution methods were coded in MATLAB 6.5 for windows. The library searches and spectral matching of the resolved pure components were conducted on the National Institute of Standards and Technology (NIST) MS database containing about 107000 compounds.

## 4. Results and Discussion

### 4.1. Resolution of the Overlapping Peaks

**Figure 1:**
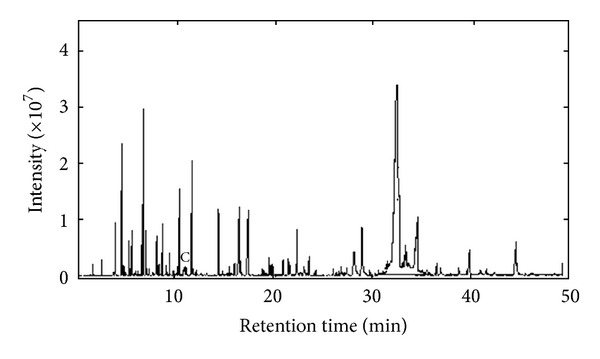
The chromatograms of the volatile components in main root of *Agrimonia eupatoria. *

The total ionic current (TIC) chromatogram of the volatile components in main root of *Agrimonia eupatoria* was shown in Figure 1 (its data matrix was denoted as **X**_1_). The intensities of the peaks recorded vary greatly. Although many chromatographic peaks are separated, here still some of eluted components overlapped, and the concentrations of some volatile components were very low. If directly searched in the NIST mass database, incorrect identification of compounds may be obtained. There were two reasons for this. First, if the chromatographic peaks were directly searched with the NIST MS database, the similarity indices (SIs) for many of these compounds were quite low. Sometimes the same component was searched at different retention time. Another reason, since peaks associated with column background and residual gases existed unavoidably in two-dimensional data obtained by mass spectral measurement, the component with low concentration was very difficult to be identified directly with the NIST mass database. However, if these overlapped peaks and the components with low content were resolved into pure spectra and chromatograms, the identification of components can be improved to a reliable extent. 

The matrix (**X**_1_) was divided into many submatrix. The chromatographic segment **X** within 11.36–11.70 min, named peak cluster C, was taken as an example. The whole procedure of this approach was demonstrated as follows.

**Figure 2:**
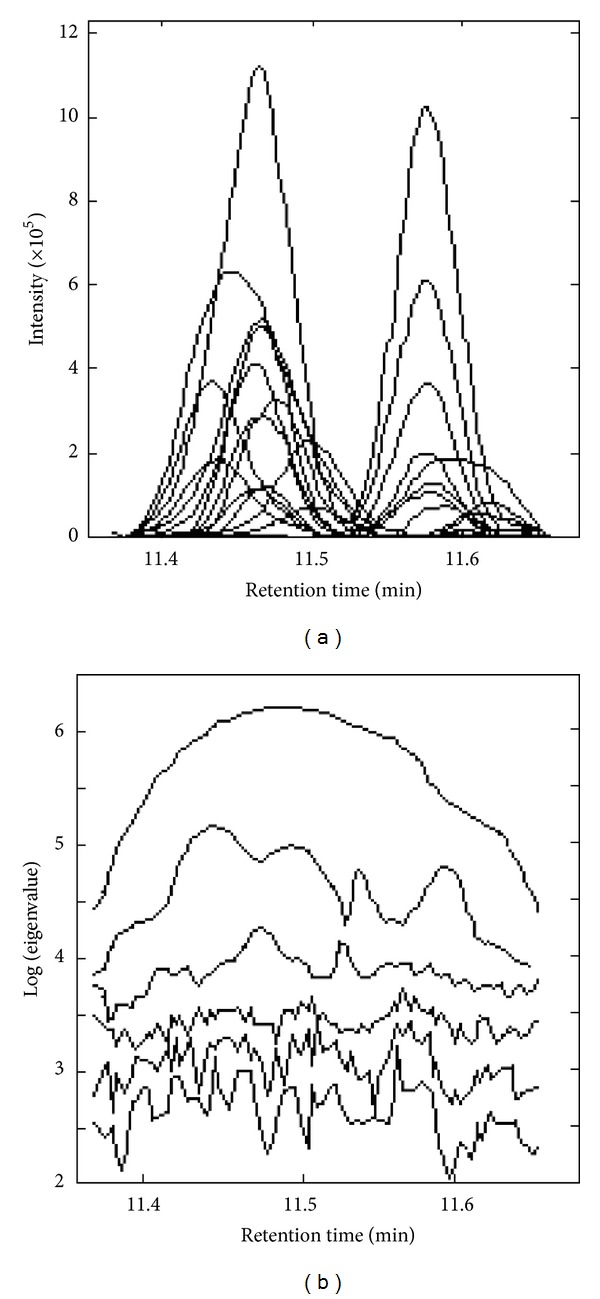
The total ion chromatogram (TIC) of the peak cluster C (a) and its rank map.

Figure 2(a) was an original chromatogram from 11.36 min to 11.70 min (peak cluster C). Intuitively there were two chemical components in this overlapping peak. However, it was impossible to get the correct qualitative and accurate quantitative results if this overlapping peak was identified directly with automatic integration and mass similarity matching provided by GC-MS workstation. The quantitative analysis of this peak cluster was also impossible, because the area of each component cannot be obtained. Here, SFA [13] was used to resolve this overlapped peak with high efficiency and accepted accuracy.

**Figure 3:**
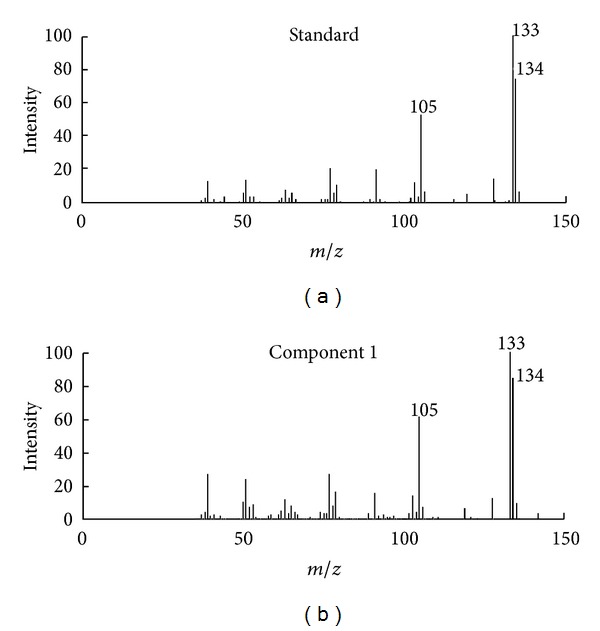
Resolved mass spectrum of component 1 by SFA and standard mass spectrum of 3,4-dimethylbenzaldehyde.

**Figure 4:**
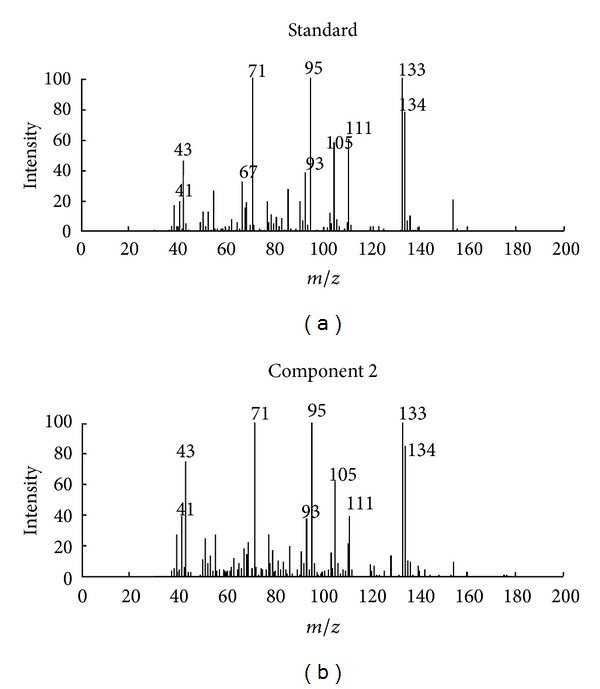
Resolved mass spectrum of component 2 by SFA and standard mass spectrum of 2,4-dimethylbenzaldehyde.

**Figure 5:**
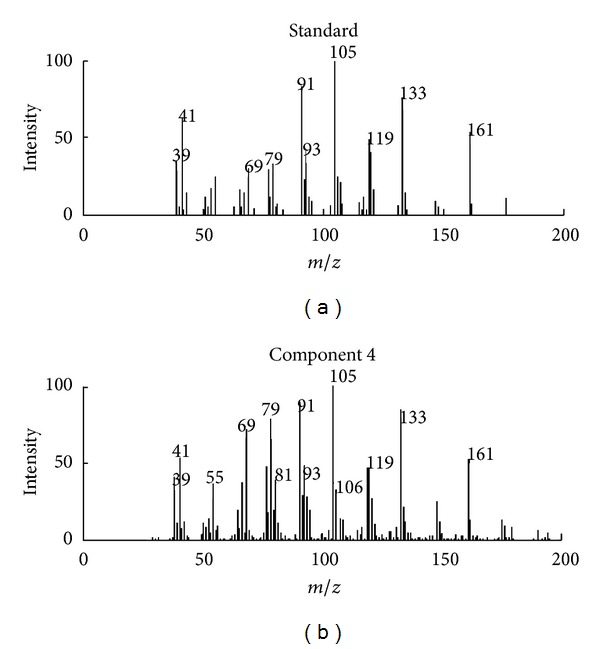
Resolved mass spectrum of component 4 by SFA and standard mass spectrum of 2-cyclopropylidene-1,7,7-trimethyl-bicyolo[2,2,1] heptane.

**Figure 6:**
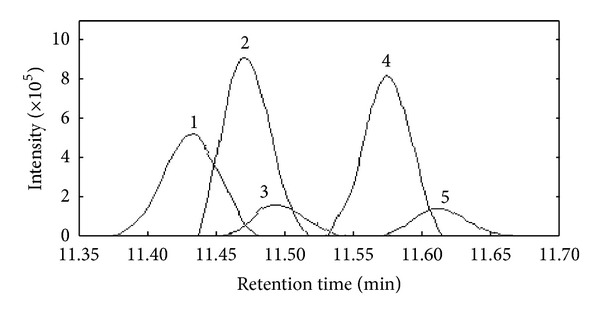
The resolved chromatogram of cluster C.

First, fix-sized moving window evolving factor analysis (FSMWEFA) was used to obtain the rank map after background correction with PCA [11]. The eluting sequences of individual components can be seen from the rank map, which was shown in Figure 2(b). A clear insight into peak cluster C was shown in the rank map. Then, the number of pure components hidden in the peak cluster and the eluting information of each component can be obtained. Determination of both left and right subwindows of each component for the use of SFA also became clear with the information mentioned above. Then, the pure spectrum of each component can be extracted by SFA directly by analyzing the correlation of two subwindows without previous resolution of their concentration profiles. The corresponding extracted mass spectrum of components 1, 2, and 4 was shown in Figures 3, 4, and 5, respectively. After all the pure spectra had been obtained, the concentration profiles could be generated by using prior information of spectra and linear regression: **C** = **X** **S**   (**S** ^*T*^ **S**)^−1^ (see (6) in Section 2.1), which were shown in Figure 6. 

### 4.2. Qualitative Analysis

Identification of the components in cluster C can be conducted by similarity searches in the NIST mass database and verified with retention indices, when each pure spectrum in cluster C was extracted and the resolved chromatographic profiles of these five components were obtained with SFA. Components 1, 2, and 4 may be 3,4-dimethylbenzaldehyde, 2,4-dimethylbenzaldehyde, and 2-cyclopropylidene-1,7,7-trimethyl-bicyolo[2,2,1] heptane, with the respective match values of 0.947, 0.967, and 0.954 (see Figures 3, 4, and 5, resp.). The match values of 0.73 and 0.68 for components 3 and 5, respectively, were too low for reliable identification of their chemical nature.

In the same way, the spectrum of each component in other segments can be obtained. Then, the corresponding identification of all the volatile components in main root of *Agrimonia eupatoria* was acquired. The qualitative results were listed in Table 1. 68 of 87 separated constituents in the total ion chromatogram of the volatile components in main root of *Agrimonia eupatoria* were identified. Comparing the obtained result with those of the literature [9, 10], this combined approach was more reliable and more components were identified satisfactorily.

### 4.3. Quantitative Analysis

Quantitative analysis was performed with the overall volume of two-way response of each component and normalization method. After all the pure chromatographic profile and mass spectrum of each component in main root of *Agrimonia eupatoria* were resolved, the total two-way response of each component can be obtained from the outer product of the concentration vector and the spectrum vector for each component, namely, **C**_*i*_ **S**_*i*_^*T*^. Similar to the general chromatographic quantitative method with peak area or height, the concentration of each component is proportional to the overall volume of its two-way response (**C**_*i*_ **S**_*i*_^*T*^). The identified components amounted quantitatively to 87.03% of the total content. The final relative quantitative results were also listed in Table 1.

### 4.4. Analysis of Correlative Components

**Figure 7:**
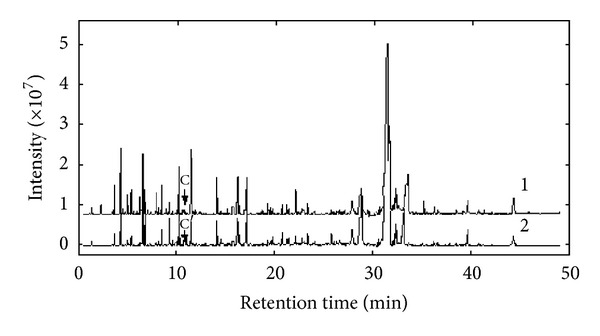
The chromatograms of the volatile components in the main root (curve 1) and leaf (curve 2) of *Agrimonia eupatoria*.

Traditional Chinese medicines (TCM) usually are very complex system. Differences maybe exist in the same Chinese herb from different areas, or different growing seasons, different plant parts of the same herb. Thus, it is very important to develop a reliable approach to analyze them. The volatile components in leaf of *Agrimonia eupatoria* have also been investigated under the same experimental conditions. Curve 1 and curve 2 in Figure 7 were the TIC chromatograms of response **X**_1_ from main root and **X**_2_ from leaf obtained from GC-MS, respectively. It was shown from Figure 7 that **X**_2_ was consistent in eluting components with **X**_1_, but the concentration distribution of some individuals was a little different. Generally, one may analyze each component in leaf of *Agrimonia eupatoria* one by one with relevant resolution method and similarity search in MS library mentioned above. However it was time-consuming to do this. The obtained information of **X**_1_ may help to reduce some arduous and unnecessary work when we compare the quality of main root and leaf of *Agrimonia eupatoria*.

Here, orthogonal projection resolution (OPR) [14] was adopted to identify each correlative component directly instead of resolving each sample data one by one with the pure component spectra in **X**_1_ resolved by SFA projecting onto sample **X**_2_. 

**Figure 8:**
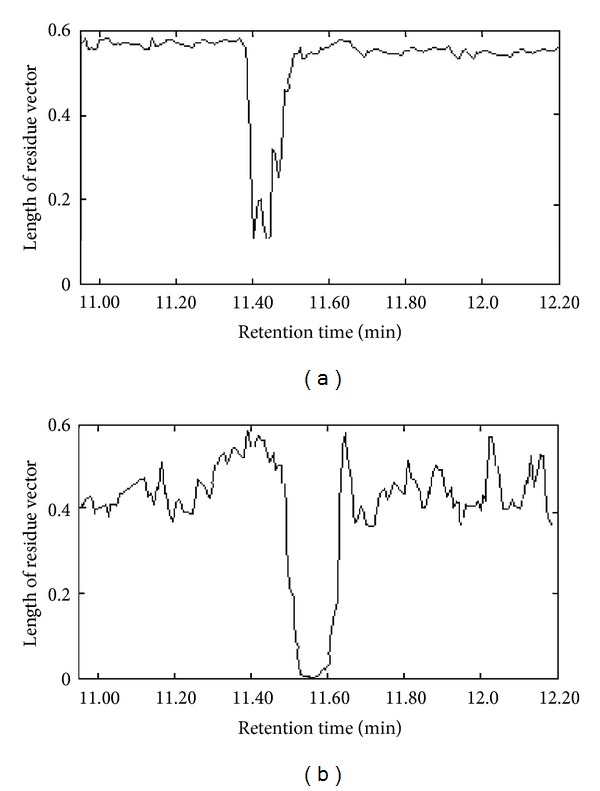
Spectrum projection graphs of component 1 (a) and 4 (b).

The chromatographic segment submatrix **X** from **X**_1_ within 11.36–11.70 min, named peak cluster C, was also used to show the procedure. As showed in Section 4.1, five components existed in it, and the pure spectrum of each component in it has been extracted with SFA. Because the retention time drift was not severe, the submatrix **X** ^**'**^ of **X**_2_, named peak cluster C′, can be selected from 11.00 to 12.20 min. Then the pure spectrum *v*_1_ and *v*_4_ of components 1 (3,4-dimethylbenzaldehyde) and 4 (2-cyclopropylidene-1,7,7-trimethyl-bicylo[2,2,1] heptane) were orthogonal projected to **X** ^**'**^. The spectrum projection graph was shown in Figure 8. It can be seen from Figure 8 that there was a range in which the length of the residue vector was close to zero to component 4. Considering the value of re_*i*_ was quite close to 0 and due to errors and interference from noise and background and so forth, in actual systems, component 2-cyclopropylidene-1,7,7-trimethyl-bicyolo[2,2,1] heptane was determined to also exist in the studied leaf of *Agrimonia eupatoria*. However, to component 1 there was not a range in which the length of the residue vector was close to zero and one can determine that component 3,4-dimethylbenzaldehyde did not exist in the studied leaf sample. Similar to this way, other correlative components, which coexisted in main root and leaf of *Agrimonia eupatoria*, could be obtained. The results were also listed in Table 1. In total, there were 52 components common to two different plant parts of *Agrimonia eupatoria*. However, because of the very low signal-to-noise ratio, some of the components may have gone undetected.

To those constituents not to be common components in the studied leaf of *Agrimonia eupatoria*, the performed procedure for main root of *Agrimonia eupatoria *was also used to extract pure spectrum of each component as described in Section 4.1. Accordingly, identification of these components were performed as in Section 4.2. Thirty components in essential oil of the studied leaf sample, which were not found in main root of *Agrimonia eupatoria *sample, were identified with SFA. The results were listed in Table 2.

### 4.5. Comparison of Samples

As shown in Tables 1 and 2, 68 and 65 volatile constituents in the main root and leaf of *Agrimonia eupatoria* were identified, respectively. Among the identified components, there were 52 common components existing in the two studied samples, but the content of each component in leaf is lower than that in main root. The main components in both main root and leaf of *Agrimonia eupatoria* were cedrol, *α*-pinene, linalool, *α*-terpineol, *α*-eudesmol, eucalyptol, and so on. The results indicated that both main root and leaf of *Agrimonia eupatoria* were consistent to some extent. By the use of similarity assessment soft, a pattern recognition program recommend by the Chinese Pharmacopoeial committee [20], the common pattern of two specimens can be constructed. The similarity of **X**_1_ and **X**_2_ to their common chromatogram was 0.9462 and 0.9417, respectively. Obviously the similarity was relatively high, but some differences also existed between them. However, components not common to both parts were generally low in abundance. The difference reflects the discrepancy between the main root and leaf of *Agrimonia eupatoria*. 

## 5. Conclusions

Combined chemometric methods were first used to analyze the volatile components in main root and leaf of *Agrimonia eupatoria*. After extraction with water distillation method, the volatile components in *Agrimonia eupatoria* were detected by GC-MS. The pure spectrum of each volatile component in main root of *Agrimonia eupatoria* was extracted with SFA. Then, OPR was used to obtain the correlative components from leaf sample. This study shows that the application of combined approach is a powerful tool, which does aim at comprehensivly revealing the quality and quantity of chemical constituents of *Agrimonia eupatoria* samples from different plant parts. The obtained information can be used for effective evaluation of similarity or differences of analytical samples. This developed method can also be used for quality control of *Agrimonia eupatoria *samples.

## Figures and Tables

**Table 1:** Identification and quantification of the volatile chemical constituents in main root and leaf of *Agrimonia eupatoria*.

Series no.	Retention time (min)		Compound name	Molecule structure	Relative content (%)
	**X**_1_^a^	**X**_2_^b^			
1	4.639	4.612	*α*-Pinene	C_10_H_16_	8.31
2	4.853	—	Hexanal	C_6_H_12_O	0.05
3	5.172	5.123	*β*-Pinene	C_10_H_16_	1.27
4	5.368	5.322	Camphene	C_10_H_16_	3.21
5	5.714	5.692	3-Octanol	C_10_H_18_O	0.27
6	6.032	5.971	Cymene	C_10_H_14_	0.18
7	6.354	6.289	D-Limonene	C_10_H_16_	1.29
8	6.632	6.617	Eucalyptol	C_10_H_18_O	3.26
9	7.290	7.195	*α*-trans-Ocimene	C_10_H_16_	0.51
10	7.572	7.547	Linalool	C_10_H_18_O	5.72
11	8.157	8.093	*α*-Campholenal	C_10_H_16_O	0.72
12	8.682	8.631	L-Camphor	C_10_H_16_O	2.11
13	9.104	—	Borneol	C_10_H_18_O	0.07
14	10.241	10.196	4-Terpineol	C_10_H_18_O	1.47
15	10.473	10.432	*α*-Terpineol	C_10_H_18_O	4.21
16	10.761	—	p-Menth-1-en-4-ol	C_10_H_18_O	0.06
17	10.941	10.906	Pulegone	C_10_H_16_O	0.17
18	11.417	—	3,4-Dimethylbenzaldehyde	C_9_H_10_O	0.41
19	11.472	11.427	2,4-Dimethylbenzaldehyde	C_9_H_10_O	0.72
20	11.576	11.512	2-Cyclopropylidene-1,7,7-trimethyl-bicyolo[2,2,1]heptane	C_13_H_20_	0.52
21	11.712	11.621	1-(2-Furyl)-1-hexanone	C_10_H_14_O_2_	4.87
22	11.801	11.762	Bergamot oil	C_12_H_20_O_2_	1.42
23	12.129	—	Nonanoic acid	C_9_H_18_O_2_	0.06
24	12.374	12.327	2-Methyl-4-hydroxyacetophenone	C_9_H_20_O_2_	0.10
25	12.871	12.821	Thymol	C_10_H_14_O	0.82
26	12.902	—	Carvacrol	C_10_H_14_O	0.44
27	13.914	13.865	Anethole	C_10_H_12_O	0.07
28	14.265	14.211	Bornyl acetate	C_12_H_20_O_2_	3.72
29	14.794	14.738	Neryl acetate	C_12_H_20_O_2_	0.47
30	14.917	14.872	Geraniol acetate	C_12_H_20_O_2_	0.61
31	15.504	—	Furan,2,5-dibutyl-	C_12_H_20_O	0.04
32	15.765	15.718	Decanoic acid	C_10_H_20_O_2_	0.06
33	16.020	15.951	Eugenol methyl ether	C_11_H_14_O_2_	0.52
34	16.812	16.762	*α*-Cedrene	C_15_H_24_	2.87
35	17.059	17.012	*α*-Longipinene	C_15_H_24_	1.42
36	17.215	17.153	Caryophyllene	C_15_H_24_	0.81
37	17.475	—	*β*-Cedrene	C_15_H_24_	0.14
38	18.176	18.093	Geranyl acetone	C_13_H_22_O	0.84
39	19.721	—	Copaene	C_15_H_24_	0.05
40	20.305	20.242	Longofolene	C_15_H_24_	0.11
41	20.437	20.381	Aromadendrene	C_15_H_24_	0.42
42	21.530	21.477	Curcumene	C_15_H_22_	0.72
43	21.875	21.813	*β*-Selinene	C_15_H_24_	0.92
44	22.041	21.872	*α*-Selinene	C_15_H_24_	0.47
45	23.057	22.971	*δ*-Guaiene	C_15_H_24_	0.61
46	23.285	—	*α*-Himachalene	C_15_H_24_	0.13
47	24.561	24.510	*α*-Bisabolene	C_15_H_24_	0.42
48	25.527	—	Acoradiene	C_15_H_24_	0.23
49	25.673	25.615	*τ*-Cadinene	C_15_H_24_	0.43
50	25.858	25.792	Cuparene	C_15_H_24_	0.37
51	26.006	25.921	Myristicin	C_11_H_12_O_3_	0.45
52	26.821	—	*α*-Guaiene	C_15_H_24_	0.09
53	27.210	27.115	trans-Nerolidol	C_15_H_26_O	0.22
54	27.708	27.647	e-Cadinene	C_15_H_24_	0.92
55	28.419	—	Caryophyllene oxide	C_15_H_24_O	0.58
56	29.275	17.292	*δ*-Cadinene	C_15_H_24_	1.53
57	31.510	31.432	Cedrol	C_15_H_26_O	14.37
58	31.576	31.425	epi-Cedrol	C_15_H_26_O	1.15
59	32.470	—	Muurolol	C_15_H_26_O	0.46
60	33.132	33.040	*α*-Cadinol	C_15_H_26_O	1.43
61	33.674	33.592	Patchoulol	C_15_H_26_O	2.17
62	34.342	—	Epiglobulol	C_15_H_26_O	0.08
63	35.027	34.631	Cubenol	C_15_H_26_O	0.72
64	37.167	37.102	Cedryl acetate	C_17_H_28_O_2_	0.76
65	39.492	39.422	Torreyol	C_15_H_26_O	0.38
66	39.861	39.784	Farnesyl acetate	C_17_H_28_O_2_	1.73
67	41.216	—	*α*-Eudesmol	C_15_H_26_O	0.06
68	44.875	44.793	6,10,14-Trimethyl-2-pentadecanone	C_18_H_36_O	1.24

^a^Representing the main root of *Agrimonia eupatoria.*

^b^Representing the leaf of *Agrimonia eupatoria.*

—: correlative component is not found in **X**_2_.

**Table 2:** Qualitative results of some other volatile chemical constituents in leaf of *Agrimonia eupatoria. *

Series no.	Retention time (min)	Compound name	Molecule structure
1	4.812	Prenal	C_5_H_8_O
2	6.359	1-Hexanol	C_6_H_14_O
3	10.717	Isomenthone	C_10_H_18_O
4	11.397	Carvone	C_10_H_14_O
5	12.105	Phenmethyl acetate	C_9_H_10_O_2_
6	12.912	4-Hydroxy-3-methylacetophenon	C_9_H_10_O_2_
7	14.493	4,4-Dimethyladamantan-2-ol	C_12_H_20_O
8	17.421	Germacrene D	C_15_H_24_
9	19.172	*β*-Damascone	C_13_H_18_O
10	25.310	Cadala-1(10),3,8-triene	C_15_H_22_
11	28.312	Longipinocarvone	C_15_H_28_O
12	32.413	Costunolide	C_15_H_20_O_2_
13	34.172	cis-7-Tetradecen-1-ol	C_14_H_28_O
14	34.247	Hexahydrofarnesyl acetone	C_18_H_36_O
